# Multiband Imaging CMOS Image Sensor with Multi-Storied Photodiode Structure †

**DOI:** 10.3390/s18061688

**Published:** 2018-05-24

**Authors:** Yoshiaki Takemoto, Mitsuhiro Tsukimura, Hideki Kato, Shunsuke Suzuki, Jun Aoki, Toru Kondo, Haruhisa Saito, Yuichi Gomi, Seisuke Matsuda, Yoshitaka Tadaki

**Affiliations:** Olympus Corporation, Tokyo 163-0914, Japan; mitsuhiro_tsukimura@ot.olympus.co.jp (M.T.); hideki_kato@ot.olympus.co.jp (H.K.); shunsuke_suzuki@ot.olympus.co.jp (S.S.); jun_aoki@ot.olympus.co.jp (J.A.); toru_kondo@ot.olympus.co.jp (T.K.); haruhisa_saito@ot.olympus.co.jp (H.S.); y_gomi@ot.olympus.co.jp (Y.G.); se_matsuda@ot.olympus.co.jp (S.M.); yoshitaka_tadaki@ot.olympus.co.jp (Y.T.)

**Keywords:** CMOS image sensor, 3D stacked, near infrared, multiband imaging

## Abstract

We developed a multiband imaging CMOS image sensor (CIS) with a multi-storied photodiode structure, which comprises two photodiode (PD) arrays that capture two different images, visible red, green, and blue (RGB) and near infrared (NIR) images at the same time. The sensor enables us to capture a wide variety of multiband images which is not limited to conventional visible RGB images taken with a Bayer filter or to invisible NIR images. Its wiring layers between two PD arrays can have an optically optimized effect by modifying its material and thickness on the bottom PD array. The incident light angle on the bottom PD depends on the thickness and structure of the wiring and bonding layer, and the structure can act as an optical filter. Its wide-range sensitivity and optimized optical filtering structure enable us to create the images of specific bands of light waves in addition to visible RGB images without designated pixels for IR among same pixel arrays without additional optical components. Our sensor will push the envelope of capturing a wide variety of multiband images.

## 1. Introduction

There has been demand for a CMOS image sensor (CIS) that captures not only visible red, green, and blue (RGB) images, but also invisible infrared (IR) images [1,2]. In the case of optical cameras, IR light is eliminated intentionally by inserting an IR cut filter in front of the surface of an image sensor to avoid color degradation. An IR signal, however, is valuable for getting additional information such as the veins lying beneath the skin because IR penetrates into deep skin where visible light doesn’t [3,4].

Conventionally, there are three methods to capture RGB and NIR mixed images at the same time; one is a method to make RGB and NIR mixed images by combining two different images taken by two CISs with a dichroic mirror, which separates visible RGB light and NIR light [5]. This combination method requires additional optical components and precise placement adjustment in order to make two images in the exact same frame. However, the required additional components increase the cost and difficulty of assembly. The second one is a method to insert an extra NIR color filter among the RGB color filter array, which means that the pixels with the NIR filter become defective pixels for RGB images [6]. This method can get RGB and NIR mixed images at the same time; however, pixel interpolation is needed to construct the RGB image. Those pixels for RGB and NIR are on the same chip, which means both pixels could not satisfy the optimized optical and electrical characteristics at the same time. The third option is to split incident light into three different wavelength regions or more by using silicon substrate as a kind of filter. Foveon demonstrated a CMOS image sensor with multi-layered different depth photodiodes in one silicon substrate [7]. This technology may make it possible to capture RGB and NIR images with one sensor and causes color degradation because of signal mixing among layered photodiodes which need a complicated device structure.

In this paper, we show the basic characteristics of the multi-storied photodiode CMOS image sensor. As a sophisticated and effective way to capture a wide variety of multiband images, in this case RGB and NIR images, at the same time without any image degradation and additional optical components, we proposed and demonstrated a multi-storied photodiode CIS with 3D stacking technology.

Section 2 describes the concept and structure of the multi-storied photodiode CMOS image sensor [8]. Section 3 presents and discusses measurement results of the basic characteristics [9]. Conclusions are presented in Section 4.

## 2. Multi-Storied Photodiode Concept and Structure

**Figure 1:**
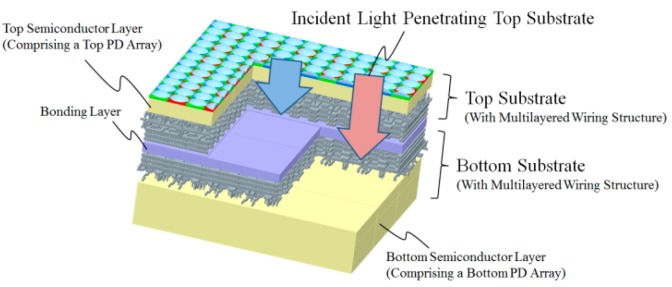
Concept of multi-storied photodiode CMOS image sensor based on 3D silicon stacked technology. The sensor comprises two layers of PD arrays, one in the top and the other in the bottom semiconductor. The top PD array converts a part of incident light into corresponding signals and works as an optical filter for the bottom PD array. The bottom PD array converts light that penetrates through the top substrate into signals, which means the top substrate acts mainly as a visible light sensor and the bottom one is an invisible IR light sensor.

Figure 1 shows a conceptual diagram of the multi-storied photodiode CMOS image sensor. The top substrate has a pixel array for mainly visible RGB light, and the bottom substrate has a pixel array for IR light that passes through the top substrate. This means that our CIS splits incident light into 6 kinds of signals, RGB signals in the top substrate, and in the bottom substrate, the other three optical signals passing through one of three color filters and the top semiconductor layer. It enables us to select specific multiband imaging by modifying the top semiconductor layer thickness or changing the characteristics of optical filters, such as the color of the filters and a multi-layered dielectric filter, on or between two substrates. Additionally, both the top and bottom substrate have their own driver circuits to adjust the driving speed and electrical shutter speed and to select read out pixels between two photodiode (PD) arrays. This function improves the dynamic range of incident light by modifying the exposure time and adapting it to the light intensity in different wavelength regions. Our novel 3D stacking technology [8,9] enables us such functions and to put the top pixels in alignment with bottom pixels to get an exact position to avoid aberrations between images obtained from the top and bottom substrates, which means no extra PDs designated for IR, which would result in a complicated fabrication process and pixels that cannot be used for visual signals would be needed on the same substrate. 3D stacking technology is also useful to achieve other functions like distance measurement or phase difference detection for lens focusing using the pixels in the bottom substrate.

**Figure 2:**
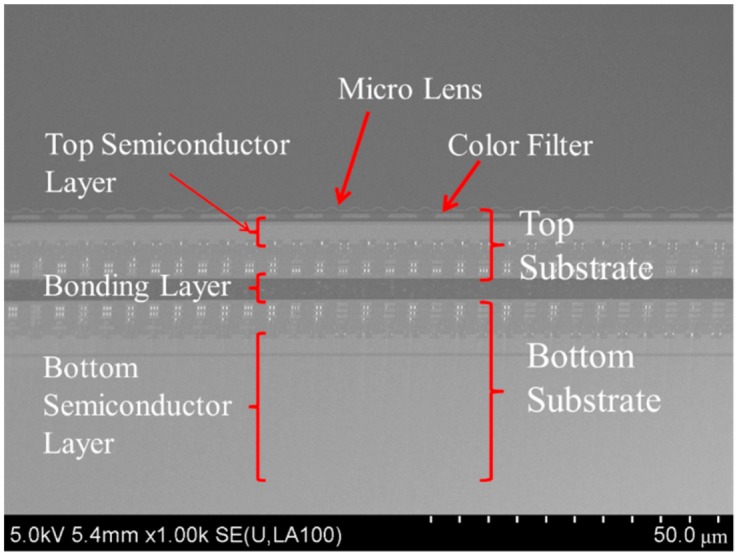
Cross-sectional SEM image of image sensor. Two substrates are bonded, and both have a semiconductor layer. Micro lenses and color filters lie on the top substrate.

Figure 2 shows a cross-sectional SEM image of the image sensor. There are micro lenses and color filters on the top PD array in the top semiconductor layer, which is 3 μm in this case. Both the top and bottom substrates have their own multilayer wiring layers to function individually. Substrates were bonded by our 3D stacked technology, which connects two substrates physically and electrically without any harm to the PDs. We measured the image sensor specification with 0.2 μm or less alignment accuracy. A part of the incident light, a longer wavelength, penetrates the top substrate and is absorbed by the bottom substrate. Incident light is absorbed by the two substrates in accordance with the wavelength so that we can have a broader range of light information and manipulate the obtained images by using two devices, e.g., we can extract IR signals from the top substrate signals with an IR reduction algorithm and IR signals from the bottom substrate to show only a visible light image. We can also show IR images from the bottom substrate at the same time.

## 3. Measurement Results and Discussion

### 3.1. Photoelectric Conversion Characteristics

**Figure 3:**
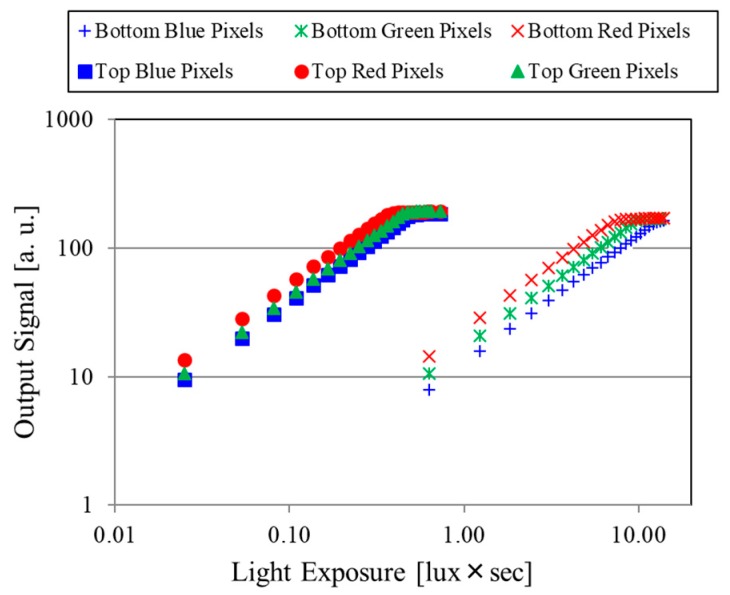
Photoelectric conversion characteristics of PDs in top and bottom substrates. Each PD array on the substrates captures incoming light properly and shows linear photoelectric conversion characteristics.

Figure 3 shows photoelectric conversion characteristics of the PDs in the top and bottom substrates with a halogen lamp, which emits a broad range of light including visible RGB light and invisible IR light. Each PD array on the substrates captures incoming light as it is designed for and shows linear photoelectric conversion characteristics. These sensitivities show the light absorption efficiency of each pixel. All PDs in the bottom substrate under both the color filters and the top substrate show linear photoelectric conversion characteristics, which means enough light passes through to be detected by the PDs in the bottom substrate.

### 3.2. Spectral Response Characteristics

**Figure 4:**
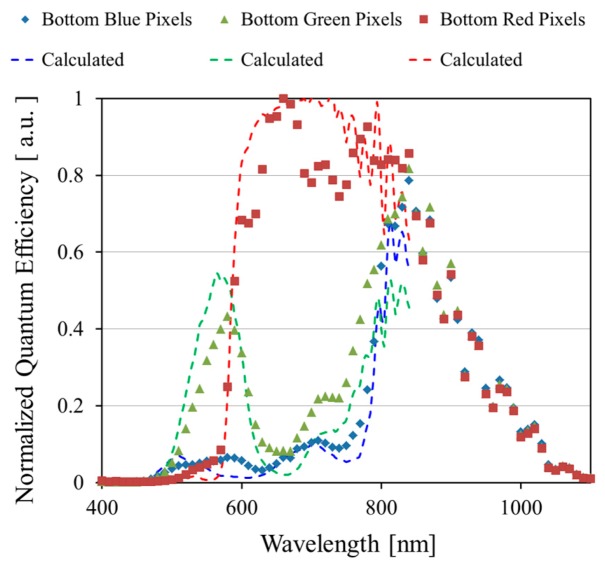
Measured and calculated normalized quantum efficiency of bottom PD array. NIR signals are detected by the bottom PD array, which comprises three types of pixels corresponding to the color filters on the top substrate.

Figure 4 shows measured and calculated normalized quantum efficiencies of the PDs in the bottom substrate; the measured data are dots, and calculated are dashed lines. Three types of color filters are arranged on the top substrate, and the incident light penetrates the filters and the top substrate. The blue, green, and red pixels in the bottom substrate showed different spectral characteristics, which means our multi-storied photodiode CMOS image sensor can obtain six kinds of spectral information. The pixels in the bottom substrate are aligned directly below the pixels in the top substrate, which means that incoming light for the pixels in the bottom substrate passes through the color filters and the top semiconductor layer. We calculated the spectral response characteristics of the bottom PDs with the optical characteristics. The measured normalized quantum efficiencies show the same tendency as our calculations with the optical constants of the filters and the substrate materials.

### 3.3. RGB and NIR Images Taken by the Multi-Storied Photodiode CMOS Image Sensor

**Figure 5:**
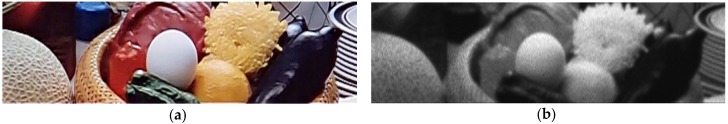
(**a**) RGB and (**b**) NIR images taken by image sensor. The RGB image was reconstructed by using both signals from PD arrays in the top and bottom substrates with IR reduction algorithm. The NIR image was obtained by using only signals from the bottom PD array.

Figure 5a shows an RGB image obtained with the signals from both substrates of the image sensor by using an IR reduction algorithm. The image does not show any color distortion without an IR filter since IR signals are subtracted adequately by the algorithm. Figure 5b shows an IR image from the bottom substrate signals taken by the same sensor at the same time. In the image, the intensity difference between pixels was adjusted by averaging the blue, green, and red pixels’ signals. These RGB and IR images, which were taken without additional optical components or additional dedicated pixels, showed no interference between the RGB image and IR signals.

**Figure 6:**
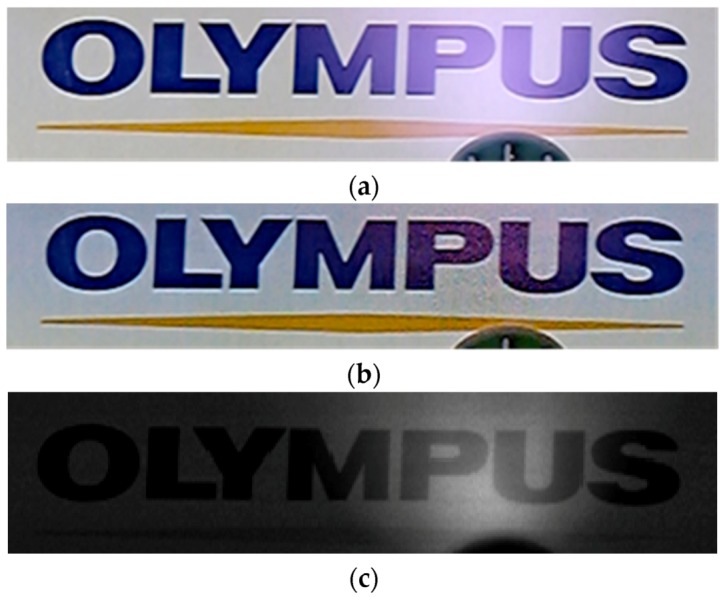
Effect of IR subtraction. (**a**) RGB image without IR subtraction. (**b**) RGB image with IR subtraction. (**c**) NIR image. The object is radiated by an NIR LED, whose wavelength and bandwidth are 900 nm and 60 nm, respectively.

**Figure 7:**
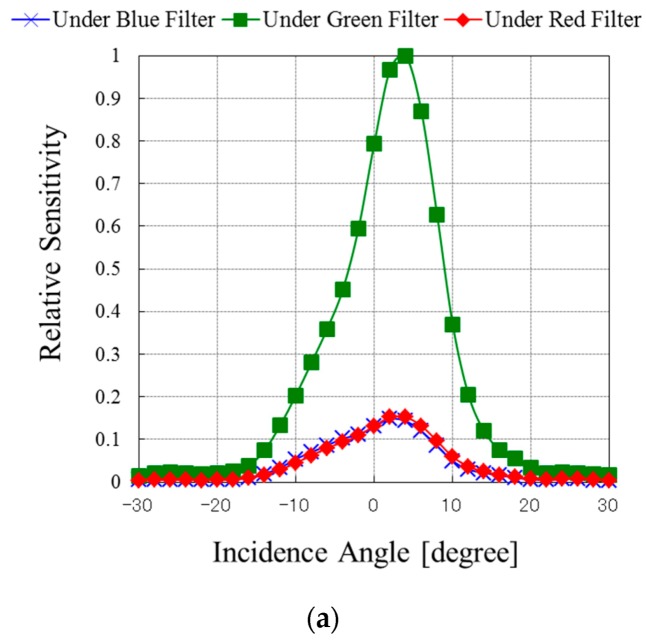

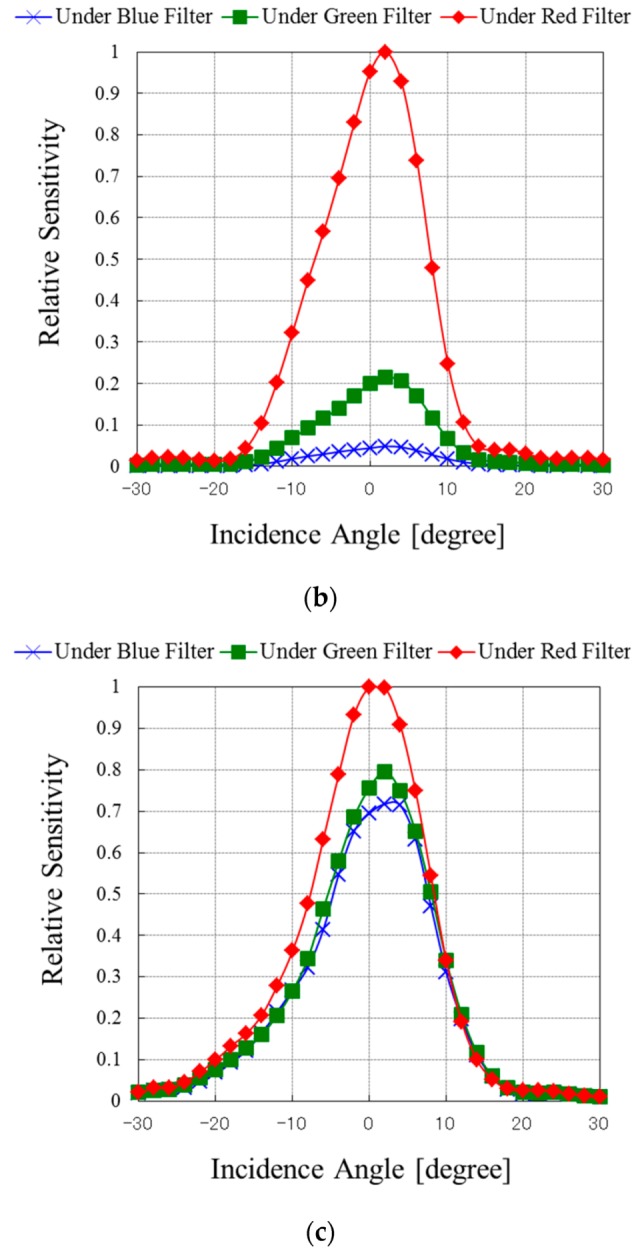
Angle dependence of normalized sensitivity of bottom PDs with (**a**) 560-, (**b**) 640-, and (**c**) 800-nm wavelength light.

Figure 6 shows the effect of IR subtraction much more clearly. We put an additional IR light emitting diode (LED), whose wavelength and bandwidth were 900 and 60 nm, respectively, as a light source to create a bright spot to degrade images. The IR subtraction algorithm was performed based on Equation (1). Here *signal_RGB_*, *signal_Top_*, and *signal_Bottom_* are the RGB signal after the subtraction, signal from the top photodiodes, and signal from the bottom photodiodes, respectively. In addition, the coefficient, A, is based on the quantum efficiencies and exposure times of the top and bottom photodiodes. The noise characteristics are described in Equation (2). This means that a bigger bottom signal decreases the signal to noise ratio of the RGB signal after IR reduction. Figure 7a shows an RGB image obtained with only the top substrate signals of a sensor. There was a bright spot caused by the extra IR light source. Figure 7b shows a RGB image with IR reduction using the bottom signals and IR reduction algorithm. Neither degradation to the color or abnormal brightness in the RGB image were caused by the extra IR light source, which means that interference to the RGB image by the extra IR was eliminated from the top substrate signals. The extra IR light can be identified as a bright spot in the image in Figure 7c. Table 1 shows the design specifications of the image sensor. The image sensor was fabricated by using a 0.18-μm 1P6M process, and the top substrate has a back-illuminated structure. The pixel size and pixel area of the multi-storied photodiodes is 3.8 μm × 3.8 μm and 16.1 mm × 0.9 mm, respectively. The photodiodes are arrayed as 4224 × 240 pixels by using our original stacking process. The top and bottom substrates were designed with the same design rule and architecture.

(1) $$Signal_{RGB}=Signal_{Top}-A\times Signal_{Bottom}$$

(2) $$Noise_{RGB}=\sqrt{{\left(Noise_{Top}\right)}^{2}+A\times {\left(Noise_{Bottom}\right)}^{2}}$$

### 3.4. RGB and NIR Images Taken by the Multi-Storied Photodiode CMOS Image Sensor

**Figure 8:**
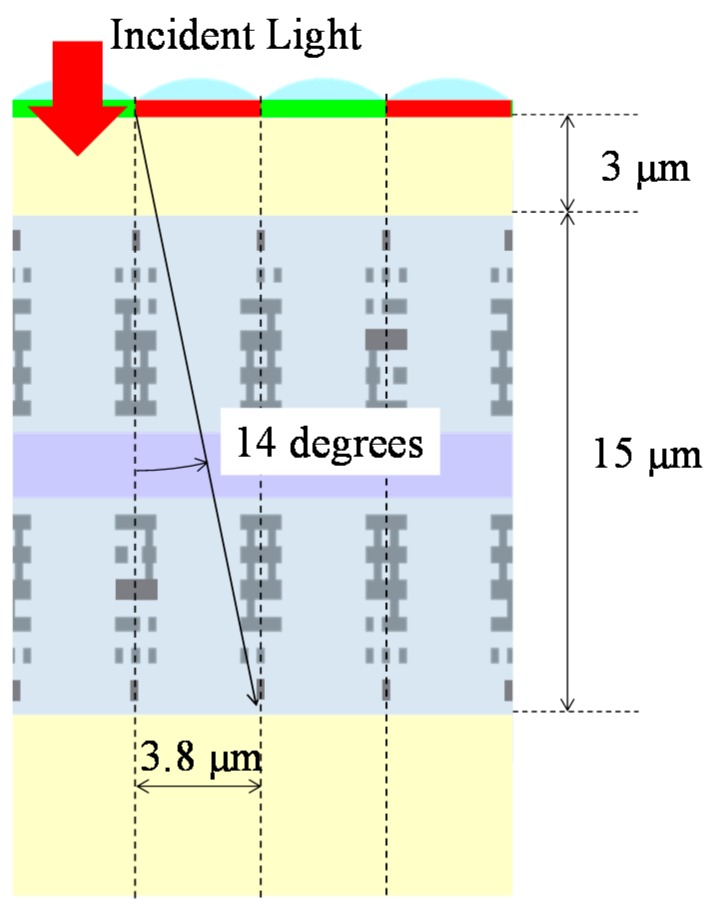
Cross-sectional schematic diagram of the multi-storied PD CIS. Incident light penetrates the top semiconductor layer, the two wiring layers, and the bonding layer and reaches the bottom PDs. Incident light is limited by the wiring layers.

Figure 7 shows the incident angle dependence of the normalized sensitivities of the PDs in the bottom substrate measured with 560-, 640-, and 800-nm wavelength light, respectively. The two substrates were aligned precisely within an accuracy of 0.2 μm, and both pixel sizes were 3.8 μm as mentioned. Their sensitivity strongly depends on the incident angle of light because the wiring layers between both substrates also work as a kind of filter and the total thickness of the wiring layers and the bonding layer was 15 μm. Figure 8 shows a cross-sectional schematic diagram of the multi-storied PD CIS. The incident light reaches the bottom PDs after penetrating the top semiconductor layer, two wiring layers, and the bonding layer. The incident light angle for the bottom PDs is less than 14 degrees because of the geometries of the wiring layouts. The measurement results show a relatively low sensitivity of over 10 degrees because the incident light is screened by the wiring layers. This means that we can design and control the incident angle characteristics of the bottom PDs by modifying the wiring layer layouts and structure along with the optical design to meet specifications. In this case, our CIS has as many as six layers of metal wiring, which limit the incident light angle as shown, and degrades the sensitivity of the bottom PDs. The quantum efficiency ratio between the top and bottom photodiode at 800 nm was 8.8% in the case of vertical incident light. Twenty percent of the incident light gets lost through the 3-μm thick silicon substrate at 800 nm and 71.2% of incident light is blocked or scattered through the wiring layers and bonding layer. The aperture ratio of wiring layers between the substrates is approximately 30%. This means incident light is refracted by the microlens and more than the aperture ratio of incident light is blocked by wiring layers. It is possible to improve the sensitivity by as much as twice by reducing the number of wiring layers to three layers in each CIS and broadening the incident light angle limit. This also suggests that modifying the material and thickness of the wiring layers between the two PD arrays can have an optical optimized effect by working as a filter for the bottom PD array.

## 4. Conclusions

We demonstrated multiband imaging and showed the characteristics of a multi-storied PD CIS which comprises two individually functioning layered devices in different substrates. The sensor was confirmed to capture a wide variety of multiband images, which is not limited to conventional visible RGB images taken with a Bayer filter or to invisible IR images, at the same time. Our device can make the conventional combination of IR and RGB imaging sensors smaller and provide a variety of multiband images. This cutting-edge multi-storied photodiode concept achieves not only multiband imaging but also other functions like distance measurement with phase difference detection for lens focusing and much more with specific pixels and circuits in both substrates. Our sensor will push the envelope of capturing a wide variety of multiband and time-divided multi-images. This article demonstrates the two layered PD CIS, however, we need to identify the absorption, blocking, scattering, and crosstalk between the substrates. An optical simulation should be performed to categorize attenuations.

## Figures and Tables

**Table 1:** Design specifications of the demonstrated multi-storied photodiode CMOS image sensor.

Items	Specifications
Fabrication process	0.18-μm 1P6M
Chip size	20.1 mm × 19.7 mm
Pixel size	3.8 μm × 3.8 μm
Multi-storied photodiode pixel area	16.1 mm × 0.9 mm
Number of multi-storied photodiodes	4224 × 240
